# Retrospective analysis of children with 46,XX testicular/ovotesticular DSD: a 10-year single-center experience

**DOI:** 10.3389/fendo.2025.1571467

**Published:** 2025-05-23

**Authors:** Yan Gong, Xiaoqin Yin, Jing Xu, Yan Li, Qingxu Liu, Shasha Zhou, Fei Wang, Yiqing Lyu, Sheng Guo, Wenyan Huang, Pin Li

**Affiliations:** ^1^ Department of Endocrinology, Shanghai Children’s Hospital, School of medicine, Shanghai Jiao Tong University, Shanghai, China; ^2^ Department of Urology, Shanghai Children’s Hospital, School of medicine, Shanghai Jiao Tong University, Shanghai, China; ^3^ Department of Nephrology, Shanghai Children’s Hospital, School of medicine, Shanghai Jiao Tong University, Shanghai, China

**Keywords:** 46, XX testicular/ovotesticular DSD, gonadal pathology, SRY gene, genetics, gonads tumor, gender assignment

## Abstract

**Purpose:**

46,XX testicular/ovotesticular differences/disorders of sexual development (TDSD/OTDSD) are rare in childhood and exhibit marked distinctions compared to those in adulthood. This study aimed to summarize the clinical characteristics and outcomes of 46,XX TDSD/OTDSD in childhood.

**Methods:**

The sexual development characteristics, hormone profiles, chromosomal analysis, fluorescence *in situ* hybridization analysis (FISH) sex-determining region Y (*SRY*) analysis (peripheral blood and tissues), molecular genetic etiology, gonadal pathology, risk of gonadal tumors, and assigned gender of 52 patients were collected and analyzed.

**Results:**

The median age at initial presentation was 18 months, and external masculinization score(EMS) within the range of 3 < EMS ≤ 6 was more prevalent. There were no statistical differences in hormone levels [luteinizing hormone (LH), follicle-stimulating hormone (FSH), and testosterone (T)] between the different age groups. Among the 52 children, 4 showed positive *SRY* in peripheral blood, whereas none of the 8 children exhibited positive *SRY* in tissue samples. A total of 29 children underwent whole exome sequencing (WES) and copy number variant (CNV) analysis, but no genetic variants were identified. A total of 47 children underwent gonadal biopsy and showed no evidence of tumors. However, immunohistochemical analysis revealed that 2 of 16 children were OCT3/4 positive. The most frequent type of gonadal pathology (17/47) was bilateral seminiferous tubules. After the assessment, gender assignment was revised in six cases: five individuals originally assigned as female at birth were reassigned as male, while one individual assigned as male was changed to female. In seven cases, the gender of rearing remained undetermined pending further longitudinal psychosocial assessment. Among the female-reared cohort, three children were more than 11 years old. As a result of undergoing bilateral gonadectomy at an early age, the patients were unable to spontaneously enter puberty. However, given their short stature, they are receiving growth hormone (GH) treatment and have not yet received sufficient sex hormone replacement therapy (HRT). Among the male-reared cohort, seven children had entered puberty. The average age at puberty onset was 12 ± 0.87 years, the average testicular volume was 5.14 ± 1.57 mL, the mean basal LH level was 6.44 ± 4.19 IU/L, the mean basal FSH level was 13.18 ± 10.22 IU/L, and the mean basal T was 3.40 ± 1.63 nmol/L.

**Conclusion:**

Compared to adults, children with 46,XX testicular/ovotesticular DSD were very different. *SRY*-negative children were predominant and tended to have more severe external genital abnormalities during childhood. Peripheral blood or tissue *SRY* mosaicism was not a prevalent cause and the intricate genetic pathways behind these cases were unknown. There were no statistical differences in hormone levels (LH, FSH, and T) between the different age groups. The assigned gender is mainly male, and the incidence of gonadal tumor risk markers was modest. During adolescence, their testosterone levels could normalize despite elevated FSH and LH levels.

## 1 Introduction

Differences of sexual development, also known as disorders of sexual development (DSD), is a complex congenital genetic disorder. Testicular tissue can develop from the XX gonads in a rare condition; this leads to a contradiction between gonadal sex and chromosomal sex (1), now known as 46,XX testicular DSD/ovotesticular DSD (46,XX TDSD/OTDSD). The literature also contains several alternative nomenclatures as well, including 46,XX male syndrome/46,XX sex reversal syndrome/46,XX sex reversal. When 46,XX male sex reversal syndrome was first proposed in 1964 (2), three distinct categories were delineated: (i) classic XX male individuals (46,XX TDSD) with normal male internal and external genitalia, but presenting infertility, gynecomastia, and sexual dysfunction in adulthood; (ii) XX male individuals (46,XX TDSD/OTDSD) with ambiguous genitalia presenting varying degrees of external genital ambiguities from birth to adolescence, such as hypospadias, micropenis, or cryptorchidism; and (iii) XX true hermaphrodites (46,XX OTDSD) presenting apparently genital ambiguities, distinguished by gonadal biopsy (3).

46,XX TDSD/OTDSD is exceedingly rare. A recent nationwide study reported that the prevalence of male individuals with 46,XX DSD reached 3.5–4.7 in 100,000 newborn male infants (4). Another study reported that testicular DSD has an estimated frequency of 1:20,000 to 1:25,000 newborn boys, while ovotesticular DSD is even rarer, with an estimated incidence of 1:100,000 births (5). Approximately 85% of cases with normal external genitalia are classified as classical and are typically diagnosed in the fields of adult urology or reproductive medicine, while only 15% of cases with genital ambiguities are identified in the pediatric center (6).

The scarcity of 46,XX TDSD/OTDSD has resulted in a paucity of documentation of cases in the pediatric field, with the majority being reported in isolated case studies or small case series. In order to address this paucity of knowledge, we conducted a retrospective analysis of 52 pediatric patients diagnosed with 46,XX TDSD/OTDSD at our center from January 2014 to June 2024. Our comprehensive evaluation included the following: clinical phenotype characterization, hormone levels, gonadal pathology, sex-determining region Y (*SRY*) analysis with fluorescence *in situ* hybridization analysis (FISH; blood and genital tissue specimens), genetic analysis related to sexual development, risk of long-term gonadal tumors, and pubertal progression.

## 2 Materials and methods

### 2.1 Subjects and clinical data

The following were the inclusion criteria (1): peripheral blood chromosomes karyotype 46,XX; (2) varying degrees of virilization; and (3) ultrasound reveals the presence of testes or gonadal pathology shows the presence of seminiferous tubules.

A total of 52 children met the inclusion criteria, and clinical data were collected, including date of birth, clinical manifestations, family history, and external masculinization score (EMS) (7). Following the administration of a series of examinations, the diagnosis of 46,XX TDSD/OTDSD was confirmed. Thereafter, patients were scheduled for follow-up visits at the center, with the frequency adjusted to 6-month intervals or annually according to their clinical status, until they reach the age of 18 years.

### 2.2 Hormone profile

In accordance with the DSD diagnostic procedure (8), the children underwent a gonadotropin-releasing hormone (GnRH) stimulation test to assess the function of the hypothalamic–pituitary–gonadal (HPG) axis in prepubertal children and to determine whether they had hyper/hypo-gonadotropic hypogonadism. At the same time, they also underwent a human chorionic gonadotropin (hCG) stimulation test to assess Leydig cell function (the function of testicular tissue in secreting testosterone).

GnRH stimulation test: Gonadorelin was administered intravenously at a dose of 2.5 μg/kg (maximum dose, 100 μg). Blood samples were collected at 0 min (baseline), 30 min/60 min/90 min post-injection to measure serum levels of luteinizing hormone (LH) and follicle-stimulating hormone (FSH). Interpretation criteria (8): Normal LH response: this is defined as a peak level occurring at 30 min post-stimulation, with the peak value exceeding threefold the baseline measurement. Blunted response: this is characterized by a peak LH increase of less than twofold compared to the baseline. Non-response: this is characterized by an absence of significant LH variation between pre-/post-stimulation samples. Delayed response: this is characterized by a peak observed at 60 min or 90 min post-stimulation. These abnormal response patterns (blunted/non-response/delayed) indicate the possibility of underlying disorders of pituitary gonadotropin secretion.

hCG stimulation test: A multiple-dose protocol was performed using an age-stratified dosing regimen: infants (500 U), childhood (1,000 U), and prepubertal stage (1500 U). Daily intramuscular injections were administered over three consecutive days. Blood samples were collected prior to the first injection and the morning following the third injection to measure serum testosterone (T) levels. Interpretation criteria (8): A normal Leydig cell response is indicated by the following post-stimulation testosterone increments relative to baseline: infancy: 2- to 10-fold increase; childhood: 5- to 10-fold increase; puberty: ≥3-fold increase.

LH, FSH, and T were completed by UniCel DxI800 (Beckman Coulter Co., Ltd., USA) through the chemiluminescence method in the laboratory department of our center.

### 2.3 Chromosomal analysis

GTG-banding at a band level of 550 following standard protocol was applied on the cultured peripheral blood cells for chromosomal karyotyping (at least 30 metaphases). We described the karyotypes according to the latest International System for Human Cytogenetic Nomenclature (ISCN).

### 2.4 Fluorescence *in situ* hybridization analysis

FISH specific for the Y chromosome was performed on 30 metaphase slides using the Vysis *SRY*/CEP X FISH Probe Kit (Abbott Laboratories, USA), and cell suspensions of peripheral blood and tissue specimens (foreskin or gonadal) were prepared according to standard procedures. Image analyses were evaluated using CytoVision software (Applied Imaging, USA) with Olympus BX61 microscope (OLYMPUS Japan). The detecting probes were as follows: red-labeled *SRY* probe and green-labeled probe for the X centromere (DXZ1).

### 2.5 Gonadal pathology and OCT3/4 by immunohistochemical assay

Gonadal biopsy was performed by a urologist in our hospital, and the biopsy specimens were sent to the pathology department of our center for hematoxylin–eosin (HE) staining. The pathological diagnoses were issued by the pathologists. OCT3/4 were performed according to standard immunohistochemical procedures (Maxin - MAB-0618, China) using a digital slide scanning device (Nano Zoomer S210, Japan) with the NDP view software.

### 2.6 Whole exome sequencing and copy number variant analysis

A standard procedure (Qiagen blood extract kit, 51106) was used to obtain genomic DNA from probands and their parents. Whole exome capture was performed using the IDT xGen^®^Exome Research Panel (IDT, USA) followed by sequencing on the HiseqX10 (Illumia, USA) platform. A core pedigree analysis was performed with parents and probands to identify single-nucleotide variants and small indels, as well as copy number variants (CNVs) of large fragments. Variants were classified for pathogenicity according to the ACMG (American College of Medical Genetics and Genomics) guideline.

### 2.7 Statistical analysis

Descriptive statistics for normally distributed variables were shown as mean with standard deviation (SD) and analyzed with *t*-test. Descriptive statistics for nonparametric data were shown as median (interquartile range, IQR) and analyzed with the Mann–Whitney *U* test or the Kruskal–Wallis test. Parameter test was used for three or more groups of normally distributed data, and Kruskal–Wallis test was used for non-normally distributed data. *P ≤ 0.05 is considered to indicate a significant difference. The statistical evaluation was conducted with the GraphPad Prism 9 program (GraphPad Software, San Diego, USA).

## 3 Results

### 3.1 Clinical presentation and hormone profile

**Figure 1:**
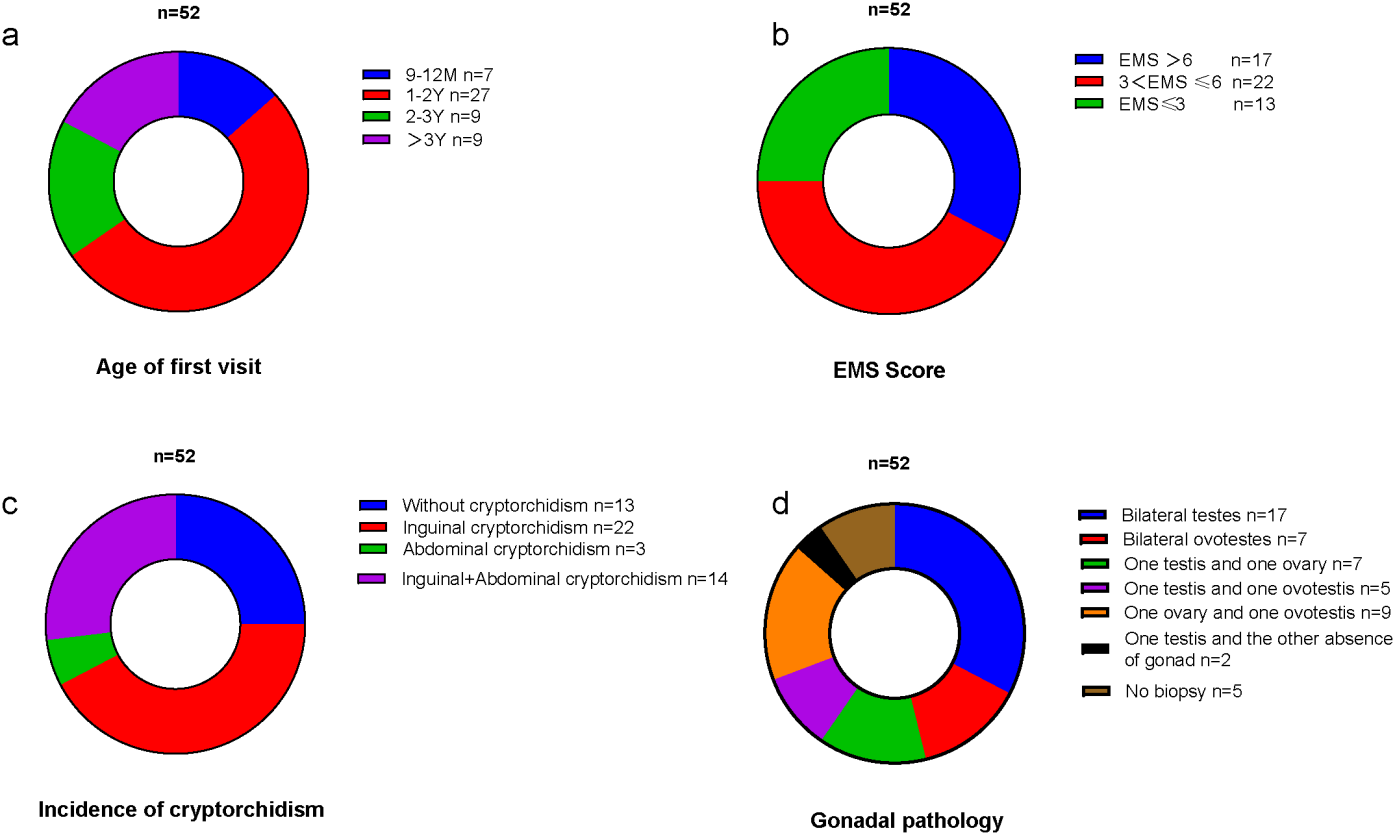
**(a)** The age distribution of the first visit. **(b)** The EMS distribution. **(c)** The incidence of different types of cryptorchidism. **(d)** The types of gonadal pathology.

A total of 52 children with 46,XX TDSD/OTDSD were included in this study. The minimum age of the first visit was 9 months, the median age was 18 months, the maximum age was 9 years, and the most common age group for the first visit was 1–2 years. The age distribution of the first visit is shown in **Figure 1a**. The basic characteristics of each patient are detailed in the **Supplementary Material**.

All children exhibited varying degrees of external genital abnormalities, including hypospadias, cryptorchidism (inguinal cryptorchidism/abdominal cryptorchidism), and micropenis. The most prevalent EMS was 3 < EMS ≤6, as illustrated in **Figure 1b**. The total incidence of cryptorchidism was 75%, with the most prevalent type being bilateral inguinal cryptorchidism (42.3%), as illustrated in **Figure 1c**. The incidence of hypospadias was 92.3%, and in four cases, the presentation was with only micropenis.

A total of 44 patients received a GnRH stimulation test at the time of initial diagnosis and did not have hypergonadotropic hypogonadism. The hCG stimulation tests were obtained in 48 children. Owing to the peculiarities of hormone levels in infants, we described in detail the clinical features of infants (*n* = 7) in this study, as detailed in **Table 1**. Then, we compared LH peak, FSH peak, and T (after hCG) in different age groups (9–12 months, 1–2 years, 2–3 years, and >3 years). The results are shown in **Table 2**, which indicates that there were no statistically significant differences in hormone levels among the different age groups.

**Table 1:** Characteristics of infantile cases (age ≤ 12 months).

Case	Initial age(months)	Gonadal pathology	LH (IU/L)	FSH (IU/L)	T (nmol/L)
1	12	One testis and one ovary	4.75	24.38	2.21
2	12	Bilateral testis	4.24	12.38	11.6
3	11	Bilateral ovotestis	2.96	11.67	7.55
4	9	Bilateral testis	2.79	10.7	/
5	12	One testis and one ovotestis	2.35	5.79	7.27
6	11	No biopsy	7.05	13	11.07
7	10	No biopsy	5.71	7.06	7.41

**Table 2:** Hormone levels between age groups.

Hormone levels	9–12 months (*n* = 7)	1–2 years (*n* = 27)	2–3 years (*n* = 9)	>3 years (*n* = 9)	*p*
LH peak (0.8–7.6) IU/L	4.26 ± 1.71	5.16 ± 2.04	3.50 ± 1.28	2.37	*p* = 0.53
FSH peak (0.7–11.4) U/L	12.14 ± 6.04	14.40 ± 7.12	13.48 ± 6.78	8.74 ± 3.93	*p* = 0.26
T (after hCG) (2.63–29.6) nmol/L	7.85 ± 3.37	6.51 ± 3.44	4.66 ± 2.16	3.84 ± 2.98	*p* = 0.07

### 3.2 FISH analysis

**Figure 2:**
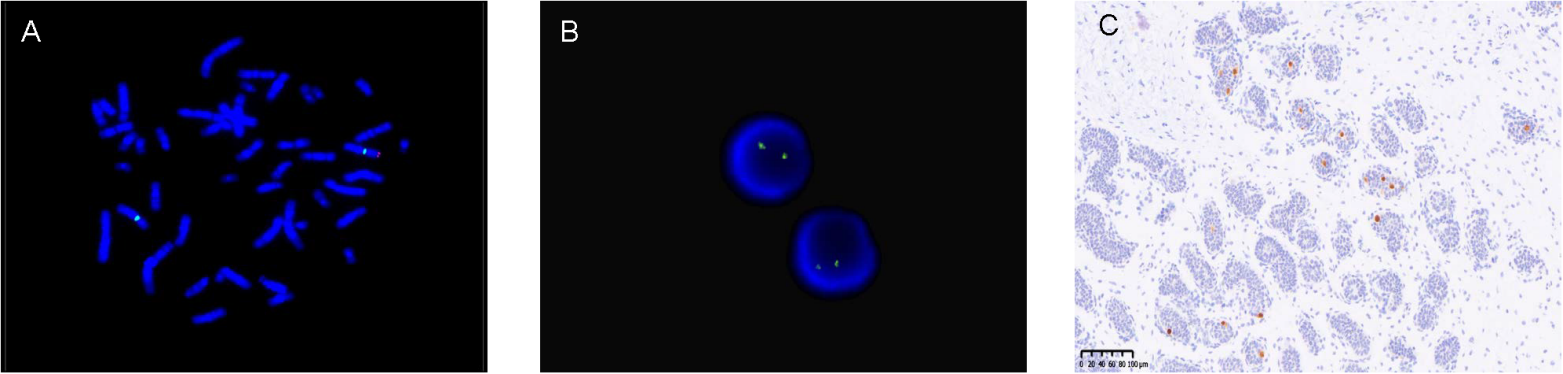
**(A)** (Peripheral blood)-FISH-two X signals (green signal) and *SRY* translocated to one of the X chromosomes (red signal). **(B)** (Gonadal tissue)-FISH-two X signals (green signal) and no *SRY* (red signal). **(C)** (Gonadal tissue)-immunohistochemistry OCT3/4 positive (brown).

All children underwent peripheral blood *SRY* detection with FISH. Of these subjects, 48 were found to be *SRY*-negative, while 4 were *SRY*-positive, indicating an instance of *SRY* translocation to an X chromosome (see **Figure 2A**). Subsequently, our molecular center carried out tissue FISH to detect *SRY* signal and eight children underwent gonadal tissue *SRY* analysis, all of whom were found to be *SRY*-negative (see **Figure 2B**).

### 3.3 Gonadal pathology

A total of 47 children underwent gonadal biopsy by the urologic surgeon to understand gonadal pathology. Individuals diagnosed with ovotesticular DSD exhibit the presence of both testicular and ovarian tissue (including bilateral ovotestes, one testis and one ovary, one ovary and one ovotestis, and one testis and one ovotestis), whereas the gonads of individuals diagnosed with testicular DSD consist exclusively of testicular tissue. In the 47 cases examined, 17 children were identified as 46,XX TDSD (bilateral seminiferous tubules), while 28 children were classified as 46,XX OTDSD. Two further cases are worthy of note. The pathological examination of one gonad revealed seminiferous tubules, while the contralateral gonad was absent and was identified as a fibrous streak-like gonad by laparoscopic exploration. The types of gonadal pathology are illustrated in **Figure 1d**.

### 3.4 Results of WES and CNV analysis

A total of 29 children underwent whole exome sequencing (WES) and CNV analysis, but no genetic variants related to sexual development were identified.

### 3.5 Gender assignment

Of the 52 children, 38 were assigned as male and 14 were assigned as female at the initial visit. Then, factors such as the child’s external genitalia condition, hormone level, gonadal pathology, family cultural background, and parents’ and children’s opinions were comprehensively considered, and then approved by the ethics committee.

The final gender assignment was as follows: five children were changed from female to male rearing gender, one child was changed from male to female rearing gender, and seven children were neutral reared. Gender assignments are shown in **Table 3**.

**Table 3:** Gender assignment.

n	Male	Female
Initial gender	38	14
Change gender	1	5
Undetermined final gender	6	1
Determined final gender	36	9

### 3.6 Gonadal management and tumor risk

The traditional treatment for ovotesticular DSD was prophylactic gonadectomy. In this study, nine children underwent early prophylactic ovotesticular gonadectomy, the oldest being 10 years and 3 months. Pathological examination of the excised gonads revealed no tumor transformation. With the understanding of the disease, our center has adopted multiple gonadal biopsies to aid in the detection of ovotestes. In addition, molecular biological marker OCT3/4 immunohistochemistry has been carried out to identify the risk of gonadal tumors at an early stage. In our study, 16 children were tested for OCT3/4, and only two children were OCT3/4(+), as shown in **Figure 2C**. With the exception of the above-mentioned children who underwent gonadectomy at an early stage, the remaining children showed no evidence of tumor manifestations at follow-up as assessed by ultrasound and tumor markers.

### 3.7 Sexual development during puberty

Among the female-reared cohort, three children were over 11 years of age. Because they underwent bilateral gonadectomy at an early age, they were unable to spontaneously enter puberty. They will receive sex hormone replacement therapy (HRT) to induce secondary sexual characteristics and maintain physiological bone mineralization. However, given their short stature, they are receiving growth hormone (GH) therapy to promote linear growth and have not yet received sufficient HRT.

Among the male-reared cohort, nine children have now reached the age of 11 years, of whom two were still prepubertal and seven had begun puberty (testicular volume ≥4mL), and two of them had gynecomastia. The maximum age of patients currently in active follow-up is 13 years and 8 months. The mean age of the seven children was 12 ± 0.87 years. The mean testicular volume was 5.14 ± 1.57 mL, the mean basal LH was 6.44 ± 4.19 IU/L, the mean basal FSH was 13.18 ± 10.22 IU/L, and the mean basal T was 3.40 ± 1.63 nmol/L. **Table 4** shows the sexual development characteristics of the seven children.

**Table 4:** Characteristics of male-raised adolescent with puberty.

Case	Current age	Gonadal pathology	Testicular volume (mL)	LH (IU/L)	FSH (IU/L)	T (nmol/L)
1	11 years, 8 months	Bilateral testis	4	4.11	5.11	2.69
2	11 years, 7 months	Bilateral testis	4	2.26	1.61	4.2
3	12 years, 3 months	One testis and one ovotestis	6	14.13	29.47	6.36
4	11 years, 1 month	Bilateral testis	4	3.66	5.73	2.53
5	11 years, 5 months	One testis and one ovotestis	6	4.08	10.48	1.17
6	12 years, 6 months	Bilateral testis	4	9.41	21.9	2.9
7	13 years, 8 months	Bilateral testis	8	7.44	17.98	3.66

## 4 Discussion

Sex determination and differentiation are processes dominated by the *SRY* gene, and several genes are expressed in an orderly and coordinated manner. Changes in any part of the process can lead to abnormal sexual development. This study is a retrospective study of the largest number of children with 46,XX TDSD/OTDSD due to the rarity of 46,XX TDSD/OTDSD.

The development of the testis is predominantly initiated by the *SRY* gene, which has been identified as the main gene responsible for the regulation of testicular determination cascade (9). 46,XX TDSD/OTDSD can be divided into *SRY*-positive and *SRY*-negative. In 1992, *SRY*-positive 46,XX male sex reversal syndrome was first reported (10). Subsequent literature reported that approximately 80% of those with the 46,XX male sex reversal syndrome were *SRY*-positive (11), and most of them are of the classical type. However, only 4 of the 52 cases in this study were *SRY-*positive, which was a very low *SRY*-positive rate and inconsistent with the literature report. This may be related to age, as the classic type often has normal male internal and external genitalia and presents to an assisted reproductive center or urology department in adulthood for infertility and poor sexual function. However, *SRY*-negative cases had been mentioned in other literature to have more severe external genital abnormalities (12), and they would usually go to the pediatric endocrinology or pediatric urology department after birth; thus, the cases diagnosed in our pediatric center showed more severe genital abnormalities, and there was a lower proportion of *SRY*-positive cases. This study confirmed that SRY-negative cases have more severe genital abnormalities: with the most common EMS is 3 < EMS ≤ 6, only four cases presented with isolated micropenis without other external genital abnormalities, the age at initial diagnosis was very young. In 2001, the possibility of *SRY* mosaicism in local tissues was proposed, and the presence of *SRY*-positive tissue in the gonads of patients with peripheral blood *SRY*-negative cases was reported (13). With the development of new technology, eight cases in our center underwent tissue *SRY* analysis. Unfortunately, no tissue *SRY*-positive cases were found. Although the biopsy site had some limitations, it also showed that *SRY* mosaicism and being *SRY*-positive are not common causes of testicular development in 46,XX individuals in childhood.

A total of 29 children underwent WES and CNV analysis, but no genetic variants were identified. We fully acknowledge the importance of identifying novel genetic findings in this study. Over the past 5 years, our team has actively collaborated with geneticists to retest the sequencing and reanalyze the patients’ sequencing data, but unfortunately, no clinically variants (pathogenic/likely pathogenic) were identified in the cohort. Although no relevant genetic variants were found, our results also suggest that common genetic variations are not a common cause of 46,XX TDSD/OTDSD. With the intricate genetic mechanisms behind testicular differentiation in 46,XX *SRY*-negative cases, we can study the pathogenesis from two main perspectives: in the absence of *SRY*, the other genes associated with testis development may be overexpressed or the activity of pro-ovarian/anti-testicular factors may be reduced. The increased expression of pro-testicular genes mainly refers to the hyper-function of genes downstream of the *SRY* pathway, such as *SOX9, SOX3, SF1, DAX1, WT1*, and *FGF9*. Among these genes, members of the *SOX* family play a significant role in this process. A number of 46,XX TDSD/OTDSD cases have been reported in the literature as a result of the *SOX* family variant. These include duplication of *SOX9* (14), duplication/triplication of *SOX9* regulatory sequences (15, 16), promoter-specific gain-of-function variant in *SOX9* (17), duplication of *SOX3* (18), rearrangement of *SOX3* regulatory sequences (19), deletion located downstream of the *SOX3* (20), and duplication of *SOX10* (21). The variants associated with the *SOX* family are the most common etiology of *SRY*-negative cases reported in the current literature. In the XX fetuses (absence of *SRY*), factors such as *NROB1, FOXL2, WNT4*, and *RSPO1* have become dominant. The upregulation of *WNT4* and *RSPO1* leads to the activation of the canonical *WNT* signaling pathway. Activation of the WNT/b-catenin pathway plays a critical role in the differentiation of the female gonad, enabling granulosa cell differentiation and ovarian differentiation (22). Decreased expression of ovarian-stimulating factors such as *RSPO1* (23), *WNT4* (24), and *NR2F2* (25) leads to testicular differentiation in 46,XX individuals. In this study, 29 children underwent WES and CNV analysis, but unfortunately, no gene variants related to sexual development were found. The mechanism of induction of testis formation in the 46,XX *SRY*-negative individual is still largely unknown. Some recent studies have focused on the involvement of epigenetic regulators in human gonadal development (26); in the ovary, the role of miRNAs in follicle assembly, growth, differentiation, and ovulation has been established (27).

It was worth mentioning that we compared hormone levels (LH peak, FSH peak, and T) between different age groups, and there was no difference between age groups. According to the literature, although testosterone levels were normal during puberty, testosterone levels were markedly decreased in adulthood, showing hypergonadotropic hypogonadism, and testicular biopsy showed severe testicular atrophy and azoospermia (12). In this study, adolescent children had normal testosterone levels, but several children had presented elevated FSH and LH, and the testicular volumes were not large enough to be consistent with the high testosterone levels, which may represent hypergonadotropic hypogonadism, as reported in the literature. This observation underscores the necessity for pediatric endocrinologists to closely monitor the functional status of the HPG axis in these individuals during their prepubertal and adolescent stages. A nationwide study showed that 73% were receiving testosterone from a median age of 19 years. Adequate testosterone replacement therapy can ensure proper masculinization with normal development of bone and muscle mass, and can also reduce the risk of diseases associated with hypogonadism (4). Individuals who are 46,XX *SRY*-negative will have infertility problems in adulthood due to the absence of the Y chromosome, including microdeletions of the AZF regions (AZFa, AZFb, AZFc, and AZFd regions), which are strongly associated with zoospermia or oligospermia (28). To date, spontaneous conception has not been reported in couples with a male partner who is 46,XX *SRY*-negative. Sperm cannot be found in 46,XX *SRY*-negative patients; thus, assisted reproductive technology using the husband’s sperm is not possible and sperm donation is more appropriate (29).

It is estimated that approximately 30% of dysplastic gonads are at risk of tumor transformation (30); thus, the traditional treatment approach was to perform prophylactic gonadectomy. In this study, nine children underwent early prophylactic gonadectomy, the oldest being 10 years and 3 months. The pathology of the excised gonads revealed no tumor transformation. It is now widely accepted that patients who are 46,XX *SRY*-negative have a low risk of tumor malignancy, even in cases of gonadal dysplasia. This phenomenon may be related to the absence of the Y chromosome (31), and the absence of the Y chromosome appears to act as an effective protective factor. However, it must be borne in mind that this conclusion may be subject to bias, given that gonadal tissues are generally removed at an early stage of life and the young age of the population studied is an obvious limitation. Consequently, patients who have undergone ovotesticular gonadal preservation should still undergo regular check-ups. The potential for bias in the sampling of a gonadal biopsy, which may result in the omission of the ovarian portion of the gonads, must be taken into consideration. A multi-point gonadal biopsy is utilized in our center with the objective of enhancing the detection rate of ovary tissue. Furthermore, the center conducts immunohistochemistry of the molecular biological marker OCT3/4 in order to identify the risk of gonadal tumors at an early stage. OCT3/4 has been proposed as a novel biomarker for the diagnosis of germ cell tumors. However, the study revealed that only 2 of the 16 patients were scattered in OCT3/4(+), of whom 1 patient had undergone positive lateral gonadectomy and the other patient was closely followed.

The timing of surgery remains a subject of controversy. In our center, prior to 2016, gonadectomy was performed early for children whose gender assignment had been decided. Research has indicated a correlation between children with DSD and a higher propensity for gender dysphoria (32). Therefore, a conservative approach was adopted in order to preserve the patient’s gonads and defer gonad removal surgery until a later age at which the patient would be in a position to consent to the procedure themselves (33). Postponing surgery may give the child the opportunity to choose their gender assignment, which may reduce their gender dysphoria. In addition, the gonads may be larger, making it easier to distinguish between the ovarian and testicular tissue, allowing for partial gonadectomy. In patients with 46,XX TDDSD/OTDSD raised as boys, ovarian tissue needs to be removed before puberty to prevent potential complications of cystic follicles due to exposure to elevated FSH and to avoid exaggerated gynecomastia due to estrogen exposure (34).

## 5 Conclusions

In summary, 46,XX TDSD/OTDSD is a very rare condition within the wide spectrum of DSD. There are very limited data on the long-term follow-up of this condition in children. We reported a cohort of patients with 46,XX TDSD/OTDSD including those who are *SRY*-negative/*SRY*-positive in our center over the past decade. 46,XX TDSD/OTDSD diagnosed in childhood was predominantly *SRY*-negative and showed more severe external abnormalities. It was very different from cases diagnosed in adulthood. Unfortunately, none of the individuals with *SRY*-negative 46,XX TDSD/OTDSD in our study had a confirmed molecular diagnosis. This highlights the possibility of unknown genetic pathways, and further research and expansion of patient cohorts are needed.

## Data Availability

The original contributions presented in the study are included in the article/**Supplementary Material**. Further inquiries can be directed to the corresponding authors.
