# Supplementary material for: Retrospective analysis of children with 46,XX testicular/ovotesticular DSD: a 10-year single-center experience
Source: Front Endocrinol (Lausanne). 2025 May 23;16:1571467. doi: 10.3389/fendo.2025.1571467 (PMC12141018; doi:10.3389/fendo.2025.1571467)
Supplement: Supplementary file 1 [file DataSheet1.pdf]

Table1. The basic characteristics of each patient.

Patient	Age at initial diagnosis	EMS	SRY (Peripheral blood)	Type on gonads	Initial gender	gender assignment
1	1y6m	7	Negative	Bilateral testes	M	M
2	3y1m	2.5	Negative	Bilateral ovotestes	**M**	**F**
3	1y1m	2	Negative	One testis and one ovary	F	F
4	12m	5	Negative	One testis and one ovary	M	M
5	9y0m	9	Positive	Bilateral testes	M	M
6	2y7m	6	Negative	One testis and one ovotestis	M	M
7	2y3m	1.5	Negative	One ovary and one ovotestis	F	F
8	4y1m	7.5	Negative	One testis and one ovotestis	M	M
9	12m	6	Negative	Bilateral testes	M	M
10	3y4m	6	Negative	Bilateral testes	M	M
11	1y4m	8	Negative	Bilateral testes	M	M
12	1y6m	6	Negative	Bilateral testes	M	M
13	2y8m	9	Negative	Bilateral testes	M	M
14	5y10m	5	Negative	Bilateral testes	**F**	**M**
15	4y5m	8	Negative	One testis and one ovotestis	M	M
16	1y1m	3	Negative	Bilateral testes	**F**	**M**
17	11m	5	Negative	Bilateral ovotestes	M	M
18	2y11m	9	Negative	Bilateral testes	M	M
19	2y9m	9	Negative	Bilateral testes	M	M
20	1y1m	2	Negative	One testis and one ovary	F	F
21	1y3m	4.5	Negative	One testis and one ovary	**F**	**M**
22	2y0m	9	Negative	Bilateral testes	M	M

23	1y2m	6	Negative	Bilateral testes	M	M
24	9m	6	Negative	Bilateral testes	M	M
25	1y2m	2	Negative	One testis and one ovotestis	**F**	**M**
26	12m	6	Negative	One testis and one ovotestis	M	Neutral
27	4y8m	2	Negative	One ovary and one ovotestis	F	F
28	1y4m	6	Negative	Bilateral ovotestes	M	M
29	1y8m	7.5	Negative	One ovary and one ovotestis	M	M
30	1y3m	7.5	Negative	Bilateral ovotestes	M	M
31	1y9m	4.5	Negative	One testis and one ovary	**F**	**M**
32	3y1m	1.5	Negative	One testis and one ovary	F	F
33	1y2m	1.5	Negative	One ovary and one ovotestis	F	F
34	1y5m	1.5	Negative	One ovary and one ovotestis	F	Neutral
35	1y7m	1.5	Negative	One ovary and one ovotestis	F	F
36	1y10m	5	Negative	One ovotestis and the other absence of gonad	M	M
37	1y2m	5	Negative	Bilateral ovotestes	M	M
38	1y6m	5	Negative	One testis and one ovary	M	M
39	2y9m	5	Negative	One ovary and one ovotestis	M	M
40	11m	6	Negative	No biopsy	M	Neutral
41	2y0m	9	Positive	No biopsy	M	Neutral
42	10m	5.5	Negative	No biopsy	M	Neutral
43	1y8m	3	Negative	Bilateral ovotestes	F	F
44	4y1m	8.5	Positive	One ovary and one ovotestis	M	M
45	1y2m	9	Negative	Bilateral testes	M	M
46	1y2m	9	Negative	Bilateral testes	M	M
47	2y4m	5	Negative	No biopsy	M	Neutral

48	1y8m	9	Positive	No biopsy	M	M
49	1y2m	6	Negative	Bilateral testes	M	M
50	2y5m	1.5	Negative	One ovary and one ovotestis	M	Neutral
51	2y7m	4	Negative	Bilateral ovotestes	M	M
52	1y2m	8	Negative	One testis and the other absence of gonad	M	M
